# Hydrogen‐Peroxide‐Generating Electrochemical Scaffold Eradicates Methicillin‐Resistant *Staphylococcus aureus* Biofilms

**DOI:** 10.1002/gch2.201800101

**Published:** 2019-03-06

**Authors:** Yash S. Raval, Abdelrhman Mohamed, Hannah M. Zmuda, Robin Patel, Haluk Beyenal

**Affiliations:** ^1^ Division of Clinical Microbiology Mayo Clinic Rochester MN 55905 USA; ^2^ The Gene and Voiland School of Chemical Engineering and Bioengineering Washington State University Pullman WA 99164 USA; ^3^ Division of Infectious Diseases Mayo Clinic Rochester MN 55905 USA

**Keywords:** biofilms, electrochemical, e‐scaffolds, H_2_O_2_, MRSA

## Abstract

Increasing rates of chronic wound infections caused by antibiotic‐resistant bacteria are a crisis in healthcare settings. Biofilms formed by bacterial communities in these wounds create a complex environment, enabling bacteria to persist, even with antibiotic treatment. Wound infections caused by methicillin‐resistant *Staphylococcus aureus* (MRSA) are major causes of morbidity in clinical practice. There is a need for new therapeutic interventions not based on antibiotics. Hydrogen peroxide (H_2_O_2_) is a known antibacterial/antibiofilm agent, continuous delivery of which has been challenging. A conductive electrochemical scaffold (e‐scaffold) is developed, which is composed of carbon fabric that electrochemically reduces dissolved oxygen into H_2_O_2_ when polarized at −0.6 V_Ag/AgCl_, as a novel antibiofilm wound dressing material. In this study, the in vitro antibiofilm activity of the e‐scaffold against MRSA is investigated. The developed e‐scaffold efficiently eradicates MRSA biofilms, based on bacterial quantitation and ATP measurements. Moreover, imaging hinted at the possibility of cell‐membrane damage as a mechanism of action. These results suggest that an H_2_O_2_‐generating e‐scaffold may be a novel platform for eliminating MRSA biofilms without using antibiotics and may be useful to treat chronic MRSA wound infections.

The recent emergence of drug‐resistant bacteria is one of the most important global health crises. According to a recent report by the World Health Organization, antibiotic resistance is a complex global public health challenge requiring more than a single strategy to address it.^1^ Infections caused by drug‐resistant bacteria may result in prolonged hospital stays and increased morbidity and mortality, compared to those caused by more susceptible bacteria.^2^ In addition to acquired resistance, biofilm‐associated resistance is a challenge in clinical practice. Bacteria, whether harboring acquired antibacterial resistance or not, can protect themselves from antibiotics through the formation of complex biofilm structures. Within biofilms, bacteria grow slowly, rendering them tolerant to many available antibiotics; further, they elaborate extracellular polymeric substances, which may provide mechanical and biochemical protection from adverse environmental conditions, including some antibiotics.^3^ Other factors rendering conventional antibiotics poorly active against microorganisms in biofilms include low pH, low oxygen availability, and low water availability within the depths of biofilms.^4^ Acquired antibacterial resistance only compounds biofilm‐associated resistance. Thus, antibiotic‐free biofilm‐directed approaches are needed to treat biofilm‐associated bacterial infections, including those caused by drug‐resistant bacteria.

Chronic wound infections represent a significant burden to patients and to the United States healthcare system overall. It has been estimated that wound infections annually affect ≈2.4–4.5 million people in United States, costing ≈$20 billion.^5^ These infections are associated with the formation of biofilms in and on the wound bed, rendering them especially challenging to treat. In addition to harboring drug‐resistant biofilms, patients with chronic wound infections typically have an excessive but ironically impaired inflammatory response, and inability of their dermal/epidermal cells to respond to repair process signals.^6^ Recalcitrant wound biofilm infections are often managed with prolonged, expensive, and often broad‐spectrum antibiotic interventions, which can contribute to long‐term development of antibiotic resistance in both targeted and nontargeted bacteria alike, and often fail outright.

One of the most common pathogens involved in chronic wound infections is *Staphylococcus aureus*.^7^ Wound infections caused by methicillin‐resistant *S. aureus* (MRSA), which has become a major challenge in chronic wound infections, especially in healthcare settings, are especially devastating because they provide a reservoir in which resistant bacteria can persist and from which they can be transmitted to others.^8^ New, nonantibiotic‐based approaches are needed to manage chronic biofilm‐associated wound infections, including those caused by MRSA.

Several approaches have been described to treat wound biofilm infections without using antibiotics. Topical antimicrobial wound dressings and electrical stimulation are some alternative methods reported to address biofilm infections and stimulate wound healing.^9^, ^10^, ^11^ Use of electrical stimulation for promoting wound healing goes back over a century ago.^12^ Electrical stimulation involves using electrodes placed near the wound surface and passing low amounts of electrical current through it. This causes disruption in the wound biofilm and promotes wound healing. It has been reported that direct electrical stimulation of wound surfaces can regulate various factors involved in the overall wound healing process, including promoting angiogenesis, increasing direct cell migration to the wound site, and decreasing edema.^13^ Use of direct current (DC) has been reported as the most common and efficient method for electrical stimulation of wound healing. Numerous in vitro and in vivo animal studies have shown enhanced wound healing with electrical stimulation. In one such study, the authors showed *Pseudomonas aeruginosa* inhibition when exposed to a DC voltage of 3.5 V.^14^ However, the authors were not able to explain the underlying mechanism of the observed antibacterial effects of electrical stimulation, attributing it to the possible production of toxic compounds. Other similar studies showed antibacterial effects of electrical stimulation to be dependent on several factors, including the type of current applied (DC or AC), type of electrode used, length of applied current and polarity of the electrodes.^12^, ^15^ However, the exact mechanism of action of electrical stimulation vis‐à‐vis wound healing remains elusive.

Frequently used topical antimicrobials and antiseptics include povidone iodine, sodium hypochlorite, chlorhexidine gluconate, silver‐/copper‐/zinc‐based dressings, medicinal honeys, and hydrogen peroxide (H_2_O_2_).^11^, ^16^, ^17^ H_2_O_2_ is of particular interest for eliminating wound biofilms and improving wound healing as low concentrations of H_2_O_2_ remove wound biofilms and stimulate wound healing.^18^, ^19^ Low concentrations of H_2_O_2_ are natural biocides commonly found in wound beds as a result of the cellular inflammatory response.^10^, ^20^, ^21^ Low amounts of H_2_O_2_ improve migration of wound‐associated fibroblasts and keratinocytes, which enhance wound healing by promoting keratinocyte differentiation.^22^ However, one of the major challenges in using an H_2_O_2_ wound dressing is that the concentration declines over time, eventually dissipating completely.^23^ To eliminate wound biofilms, wound scaffolds that continuously produce and deliver antimicrobial agents such as H_2_O_2_, without resulting in toxicity to human cells, are needed.

Previously, we developed and studied the anti‐biofilm property of an H_2_O_2_‐generating electrochemical scaffold (e‐scaffold) with biofilms of *Acinetobacter baumannii* and *Pseudomonas aeruginosa* and also determined its biocompatibility with mammalian tissue.^19^, ^24^ In this work, we report application of the H_2_O_2‐_producing e‐scaffold as a novel antibiotic‐free approach to eliminate MRSA biofilms and also explore the potential anti‐biofilm mechanism of the H_2_O_2_‐generating e‐scaffold. The H_2_O_2_ e‐scaffold offers numerous advantages over traditional antibiotic/antiseptic treatments in that it can be designed in any shape, which is important to cover entire wound surfaces; it can target a diversity of biofilm‐forming pathogens present in wounds; and, since it relies on production of H_2_O_2_ to inhibit biofilm‐related infections, it minimize selection of bacterial strains resistant to traditional antibiotics. We designed our e‐scaffold of conductive carbon fabric due to its flexibility and biocompatibility.^19^, ^25^ The e‐scaffold development is based on our previous work.^19^ Our e‐scaffold contains three electrodes; a working electrode (circular carbon fabric patch having area of 6.42 cm^2^), which has negative polarity and can reduce dissolved oxygen in wound bed (or liquid phase) and generate H_2_O_2_; a counter electrode (a smaller circular carbon fabric patch having area of 2.14 cm^2^) and a customized Ag/AgCl reference electrode (**Figure** **1**). Additional assembly details of the e‐scaffold can be found in our previous study.^19^ Briefly, both the larger carbon fabric patch (acting as a working electrode) and smaller carbon fabric patch (acting as a counter electrode) are attached to each other using a silicone rubber sealant, which acts as an insulating agent between the electrodes while allowing oxygen to freely diffuse through the entire e‐scaffold surface for H_2_O_2_ production. Titanium wires are attached onto both side‐ends of e‐scaffold via nylon sew snaps. Titanium wires are connected to external cables that in turn are connected to custom‐made potentiostat.^26^ The electric potential applied to the e‐scaffold is carefully controlled via a potentiostat. Based on our previous studies, the surface of the e‐scaffold was polarized at −0.6 V_Ag/AgCl_ for continuous production of H_2_O_2_.^19^, ^24^, ^27^ Under these conditions, we observed constant production of H_2_O_2_ (≈25 × 10^−6^ m concentration) at the e‐scaffold surface, consistent with our previous studies.^27^, ^28^ It was also observed that the amount of H_2_O_2_ produced on the surface of the e‐scaffold was dependent on the applied voltage. We detected H_2_O_2_ on the scaffold surface when it was polarized below −0.3 V_Ag/AgCl_; the maximum concentration of H_2_O_2_ (≈25 × 10^−6^ m near the e‐scaffold surface) was produced when the potential was −0.6 V_Ag/AgCl_.^19^ Several studies have reported that this concentration of H_2_O_2_ does not cause observable toxicity to mammalian tissues and that may actually promote wound healing.^21^, ^29^

**Figure 1:**
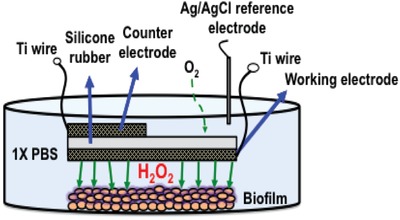
Schematic representation and assembly of H_2_O_2_‐generating e‐scaffold. The working, counter, and reference electrodes were connected to a potentiostat.

To determine the biofilm eradicating property of our H_2_O_2‐_producing e‐scaffold against MRSA strains when polarized at −0.6 V_Ag/AgCl_, three strains of MRSA (USA100, USA200, and USA300) were allowed to form fresh biofilms in a six‐well plate for 24 h (Supporting Information). After 24 h, the biofilm was washed thrice with sterile phosphate buffer saline (PBS) and resuspended in 7 mL of PBS. A sterile e‐scaffold was carefully placed on top of the MRSA biofilm layer making sure that the biofilm was not disturbed. The wiring of the e‐scaffold was connected to a potentiostat and the working electrode polarized at −0.6 V_Ag/AgCl_ after conditioning the e‐scaffold in PBS using cyclic voltammetry.^19^ Later, the biofilm was exposed to polarized e‐scaffold treatment for 6, 12, and 24 h. Control biofilms experiments consisted of nonpolarized e‐scaffold treatment. After treatment, biofilm cells were scraped from the glass‐well surface as well as from e‐scaffold surface and these suspensions were vortexed and centrifuged (4000 rpm; 10 min). The cell pellet was resuspended in 1 mL of PBS and serial dilutions were prepared. 100 µL of each dilution was spread plated onto blood agar plates. Plates were then incubated at 37 °C for 48 h, after which colony‐forming units (CFU) were counted and data reported as log_10_ CFU cm^−2^. **Figure** **2** shows the reduction in CFU counts of MRSA biofilms after e‐scaffold treatment. As evident from Figure 2, all tested MRSA strains exposed to the polarized e‐scaffold showed significant reductions in CFU counts compared to controls, which showed no reduction in CFU counts (*p* < 0.01). The reduction in CFU counts of MRSA biofilms was dependent on e‐scaffold exposure time. After 24 h treatment with the H_2_O_2_‐producing e‐scaffold, ≈4‐log reduction in MRSA biofilm cells was achieved (*p* value < 0.001); 12 h exposure to the e‐scaffold reduced CFU counts of MRSA biofilms by ≈3‐logs (*p* value < 0.001) and 6 h exposure resulted in ≈2‐log reduction (*p* value < 0.01). Interestingly, we did not observe any differences in MRSA biofilm reduction among the three study strains.

**Figure 2:**
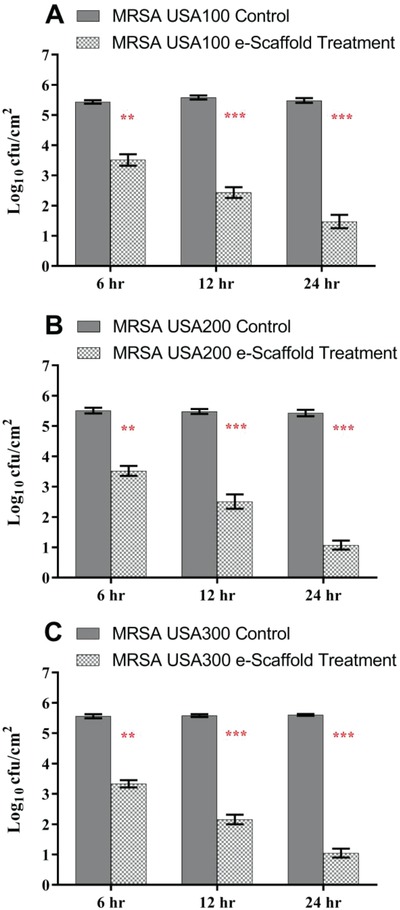
CFU cm^−2^ counts of A) MRSA USA100; B) MRSA USA200; and C) MRSA USA300 biofilms after e‐scaffold treatment for 6, 12, and 24 h compared to untreated controls. Data are represented as means ± SD (*n* = 3). Statistical analysis was performed by one‐way ANOVA (**p‐value < 0.01; ***p value < 0.001).

Among various biocides currently used in clinical settings, H_2_O_2_ has been recognized for disinfection and sterilization due to its ability to form reactive oxygen species (ROS). Moreover, usage of up to 3% H_2_O_2_ (980 × 10^−3^ m) in healthcare settings has been approved by the US Food and Drug Administration.^21^, ^30^ Bacteria present in biofilms show more resistance to biocides, disinfectants and antibiotics compared to their planktonic forms.^31^ Hence, using high concentrations of such biocides is a common way to eradicate biofilms. In one study, direct incubation of H_2_O_2_ (5%) on growing biofilms under in vitro conditions decreased the viability of *Staphylococcus epidermidis* biofilms.^32^ Another study revealed that 3% H_2_O_2_ decreased the biofilm‐forming capability of clinically isolated *S. aureus* strains.^33^ However, it should be noted that that high concentrations of H_2_O_2_ could be detrimental to mammalian tissues as H_2_O_2_ can cause oxidative damage. In this regard, our e‐scaffold system is unique in that it continuously produces low concentration of H_2_O_2_, enabling biofilm eradication while minimizing toxicity to tissues.^21^, ^29^, ^34^

H_2_O_2_ belongs to the broader group of reactive oxygen species, which also includes hydroxyl free radicals (OH·), singlet oxygen (^1^O_2_) and superoxide radicals (O_2_^−^). H_2_O_2_ can enter into the bacterial membrane and initiate intracellular production of other reactive oxygen species that can cause oxidative stress.^35^, ^36^ These reactive oxygen species can interact with and degrade bacterial DNA, RNA, proteins, and cell‐membrane lipids.^36^, ^37^ To explain the potential mechanism through which H_2_O_2_ produced by our e‐scaffold interacts with the bacteria present in MRSA biofilms, we performed a live/dead bacterial cell‐membrane staining assay to qualitatively assess membrane integrity after e‐scaffold treatment. After exposing freshly grown MRSA USA100 biofilm to the H_2_O_2_‐producing e‐scaffold for different time periods, biofilms were stained with a mixture of SYTO 9 and propidium iodide dyes (Supporting Information) and observed under confocal laser scanning microscope. SYTO 9 is a green‐fluorescent membrane permeable dye that stains both live and dead bacterial cells. In contrast, propidium iodide, a red fluorescent dye, can only enter bacterial cells if they have damaged cell membranes. **Figure** **3** shows the results of live/dead staining of MRSA USA100 biofilm after treatment with the e‐scaffold. Figure 3A,D,G shows the overall amount of live cells present in the MRSA USA100 biofilm after exposing it to the H_2_O_2_‐producing e‐scaffold for 6, 12, and 24 h, respectively; the overall amount of live cells (stained green) present in the biofilm decreased with an increase in time of e‐scaffold exposure. Figure 3B,E,H shows the overall amount of dead cells present in the MRSA USA100 biofilm after exposing it to the H_2_O_2_‐producing e‐scaffold for 6, 12, and 24 h, respectively; these images demonstrate that the overall amount of dead cells (stained red) present in the biofilm increases significantly with increasing time of e‐scaffold exposure. Conversely, the control group of MRSA USA100 biofilms (i.e., exposed to a nonpolarized e‐scaffold) did not show any significant amount of dead cells (Figure S1, Supporting Information). Based on the confocal laser scanning microscopy images, polarized H_2_O_2_‐producing e‐scaffold treatment of MRSA USA100 biofilm may have resulted in disruption of cell membranes within the biofilm. As propidium iodide is a membrane impermeable dye, it can only cross bacterial membranes if their structural integrity has been compromised. Based on the results of the live/dead staining assay, the H_2_O_2_‐producing e‐scaffold appears to damage the membranes of bacteria present in biofilms. H_2_O_2_, along with other members of reactive oxygen species [e.g., hydroxyl free radicals (OH·)] can damage membranes, potentially facilitating entry of propidium iodide into cells.^24^ Several recent studies have shown that H_2_O_2_‐induced intracellular production of hydroxyl free radicals (OH·) can destroy persister cells present in biofilms, resulting in biofilm dispersal and also disrupt global biofilm structures in Gram‐negative bacteria by increasing bacterial membrane permeability.^37^, ^38^ In a recent study, e‐scaffold generated H_2_O_2_‐induced intracellular production of hydroxyl free radicals (OH·) increased membrane permeability of *P. aeruginosa* biofilms as observed through increased fluorescence of propidium iodide, suggesting that membrane disruption via production of ROS, of which hydroxyl free radicals (OH·) is a member, may be one of the mechanisms through which the e‐scaffold exerts its antibiofilm effect.^24^ Since propidium iodide also stains extracellular DNA present in the biofilm matrix, additional quantitative analysis of biofilm biomass matrix through 3D reconstruction of confocal images is warranted for accurate quantification of live and dead cells present in biofilm since the loss of biomass may be due to rinsing away dispersed cells prior to imaging. Further mechanistic and enzymatic studies are needed to determine the overall mechanisms through which H_2_O_2_ produced from our e‐scaffold kills bacterial cells in biofilms.

**Figure 3:**
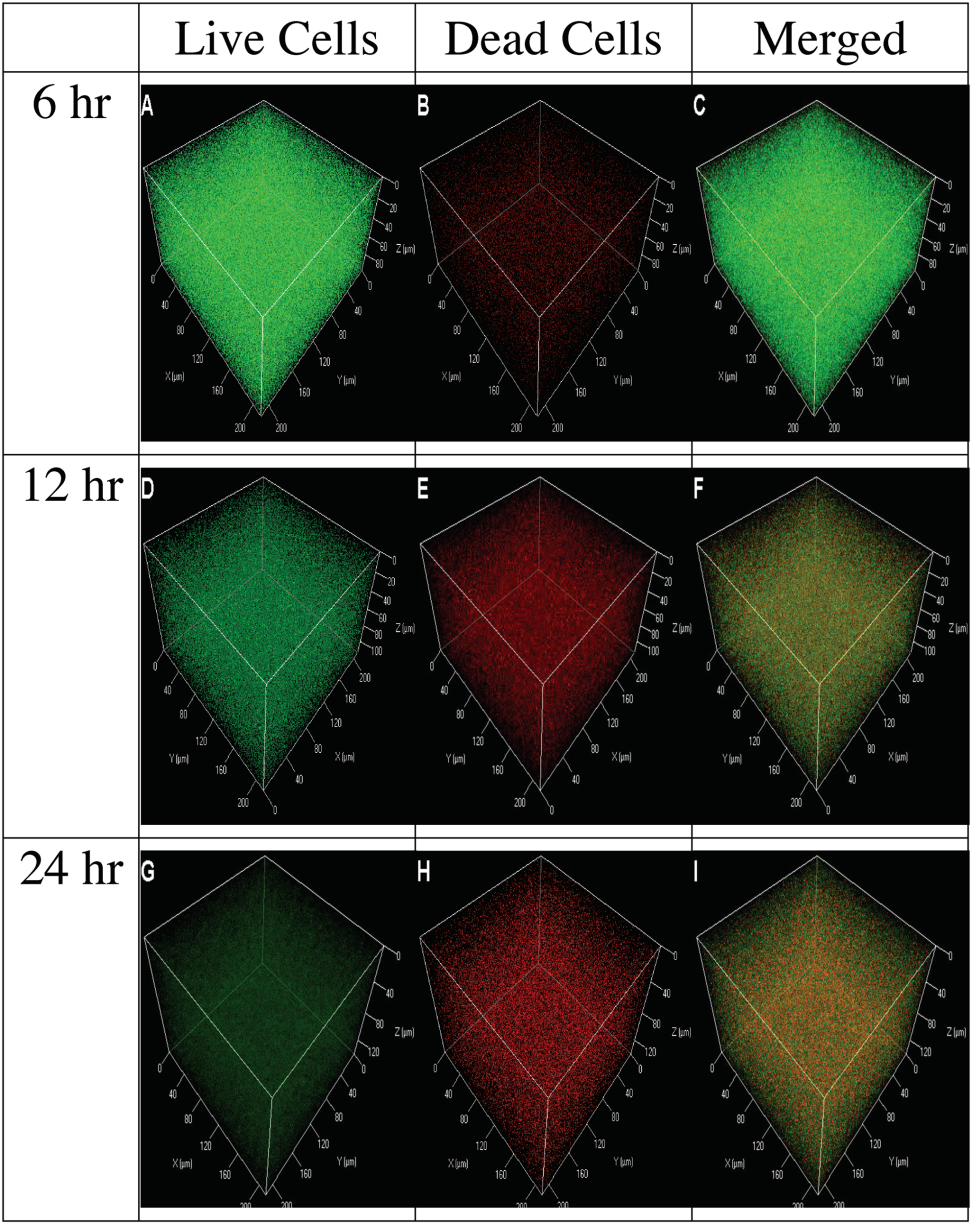
Live/dead staining of MRSA USA100 biofilms after e‐scaffold treatment. MRSA USA100 biofilms exposed to e‐scaffolds for 6, 12, and 24 h were stained with a mixture of SYTO 9 and propidium iodide dyes (causing live cells to appear green and dead cells red). Total magnification: 400×.

Intracellular adenosine triphosphate (ATP) levels in bacterial cells generally determine the cells' metabolic state. Any sudden changes in external environmental factors, such as temperature, carbon source, nutrients, stress, or toxic chemicals, can directly affect intracellular ATP levels. The presence of toxic chemicals/compounds that are detrimental to bacterial cells can considerably decrease cellular ATP levels. Presently, some antibiotics used in clinical settings for treating MRSA infections interact with bacterial cell membrane components as part of their antibacterial mechanisms.^39^ Because only metabolically active and viable bacterial cells constantly produce ATP, we selected a luciferase‐based ATP assay as a biofilm viability marker assay. Since the presence of reactive oxygen species (which include H_2_O_2_) exert oxidative stress on bacterial cells, we conducted an ATP assay based on luminescence to determine the effect of our H_2_O_2_‐producing e‐scaffold on bacterial energy metabolism and overall intracellular ATP levels of bacterial cells present in MRSA USA100 biofilms after exposure to e‐scaffold treatment. The ATP assay was carried out according to manufacturer's protocol with a small modification (Supporting Information). Freshly grown MRSA USA100 biofilms were exposed to our H_2_O_2_‐producing e‐scaffold for 6, 12, and 24 h. After exposure, the intracellular ATP levels of cells present in MRSA USA100 biofilms were measured. As seen in **Figure** **4**, there was a marked decrease in ATP levels after exposure to e‐scaffold treatment (*p* value < 0.0001). Moreover, the reduction in ATP levels was found to be dependent upon the time the biofilm was exposed to e‐scaffold treatment. Interestingly, the control group biofilm (exposed to a nonpolarized e‐scaffold) did not show any decrease in the overall ATP levels. Similar decreases in ATP levels was reported when *S. aureus* biofilms were treated with different types of New Zealand Manuka honey.^16^ Interestingly, the authors of that study also claimed that the presence of H_2_O_2_ and methylglyoxal in honey was partly responsible for the reduction in ATP levels in *S. aureus* biofilms. Another study found a significant decrease in ATP levels of MRSA biofilm when exposed to various bacteriocins.^40^ A possible mechanisms to explain the large decrease in ATP levels observed in our study is an increase in oxidative stress exerted by H_2_O_2_. This can diminish the cell membrane potential of bacteria, which can lead to interruptions in the working machinery of ATP synthase pumps. This can in turn reduce proton motive force, cause membrane depolarization and ultimately result in cell death. Future studies are required to further explain the exact mechanism as to how H_2_O_2_ produced by our e‐scaffold interacts with and interferes with various carbohydrate and enzymatic systems present in the electron transport system of bacterial cells.

**Figure 4:**
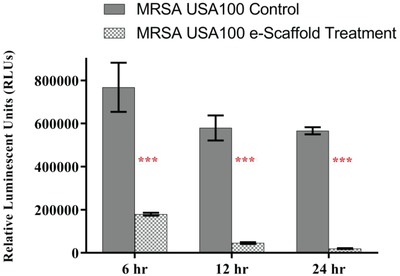
Intracellular ATP levels of MRSA USA100 biofilms with e‐scaffold treatment for 6, 12, and 24 h compared to untreated controls. Data are represented as means ± SD (*n* = 3). Statistical analysis was performed by one‐way ANOVA (***p value < 0.001).

One limitation of this study is that we did not compare our results to those achievable with conventional antibiotics. There are several reasons for this, first and foremost of which is that our goal was to specifically provide an antibiotic‐free approach. Further, any true comparison to conventional antibiotics would have required testing a suite of compounds representing different classes of agents and at varying concentrations. We note that in previous studies, we showed that our e‐scaffold kills persister cells, which are not killed by antibiotics.^24^ Another limitation is that we did not include an exogenous H_2_O_2_ control; however, we did do this in our prior work.^19^, ^24^

In conclusion, we demonstrate that electrochemically generated H_2_O_2_ using a novel e‐scaffold can potentially act as novel antibiotic‐free platform to eradicate MRSA biofilms. By polarizing the e‐scaffold at −0.6 V_Ag/AgCl_, we continuously generated H_2_O_2_ at low concentrations, sufficient to kill biofilms. Exposure to the H_2_O_2_‐producing e‐scaffold resulted in significant reduction in colony counts of MRSA biofilms. This reduction was dependent on exposure time; after a 24 h exposure, we observed ≈4‐log reduction in viability of MRSA biofilms regardless of type strain. Structural cell membrane integrity was compromised in biofilms of MRSA USA100 exposed to the e‐scaffold. Moreover, the overall intracellular ATP levels of MRSA USA100 biofilms were decreased in the presence of e‐scaffold treatment. We propose based on these results that a potential mechanism through which the described e‐scaffold interacts with bacteria is through interaction with the cell membrane and its structural and biochemical components. These results suggest that an H_2_O_2_‐producing e‐scaffold could be an alternative to conventional antibiotics for chronic wound biofilm infections. Future studies will include a detailed investigation as to the antibiofilm activity of our e‐scaffold, assessing its activity in a mixed‐species biofilm environment, quantitative analysis of 3D reconstructed biofilm images after e‐scaffold treatment and future in vivo studies evaluating efficacy, biocompatibility and safe usage.

## Conflict of Interest

RP reports grants from CD Diagnostics, BioFire, Curetis, Merck, Hutchison Biofilm Medical Solutions, Accelerate Diagnostics, Allergan, and The Medicines Company. consultant to Curetis, Qvella, St. Jude, Beckman Coulter, Morgan Stanley, Heraeus Medical GmbH, CORMATRIX, Specific Technologies, Diaxonit, Selux Dx, GenMark Diagnostics, LBT Innovations Ltd, PathoQuest and Genentech; monies are paid to Mayo Clinic.

## Supporting information

Supplementary
