# Supplementary material for: Hydrogen‐Peroxide‐Generating Electrochemical Scaffold Eradicates Methicillin‐Resistant Staphylococcus aureus Biofilms
Source: Glob Chall. 2019 Mar 6;3(6):1800101. doi: 10.1002/gch2.201800101 (PMC6551415; doi:10.1002/gch2.201800101)
Supplement: Supplementary file 1 — Supplementary [file GCH2-3-1800101-s001.pdf]

Copyright WILEY-VCH Verlag GmbH & Co. KGaA, 69469 Weinheim, Germany, 2019.

Global Challenges

Open Access

Supporting Information

for *Global Challenges,* DOI: 10.1002/gch2.201800101

Hydrogen-Peroxide-Generating Electrochemical Scaffold Eradicates Methicillin-Resistant *Staphylococcus aureus* Biofilms

Yash S. Raval, Abdelrhman Mohamed, Hannah M. Zmuda, Robin Patel, and Haluk Beyenal**

Supplementary Information

Hydrogen Peroxide Generating Electrochemical Scaffold For Eradication of Methicillin-Resistant Staphylococcus aureus Biofilms

Yash .S. Raval[a], Abdelrhman Mohamed[c], Hannah Zmuda[a,c], Robin Patel[*a,b], Haluk Beyenal[*c]
[a]Division of Clinical Microbiology, Mayo Clinic, Rochester, MN 55905, USA
[b]Division of Infectious Diseases, Mayo Clinic, Rochester, MN 55905, USA
[c]The Gene and Voiland School of Chemical Engineering and Bioengineering, Washington State University, Pullman, WA 99164, USA

Preparation of MRSA biofilms: Overnight cultures of methicillin-resistant Staphylococcus aureus (MRSA) strains USA 100, USA200 and USA300 were grown in tryptic soy broth (TSB) supplemented with 1% glucose. ~ 1×10^4 CFU/ml of fresh culture (based on OD reading/quantitative culture) was added to 2 ml of TSB supplemented with 1% glucose in individual wells of a 6-well plate. The plate was incubated at 37°C for 24 hr (without shaking) to allow for biofilm growth. Next, the biofilm was washed with 1X phosphate buffer saline (PBS) 3 times to remove planktonic bacteria and finally resuspended in 7 ml of PBS.

Exposure of e-scaffold to MRSA biofilms: A sterile e-scaffold was carefully placed on top of the biofilm layer making sure that the biofilm was not disturbed. The wiring of the e-scaffold was connected to a potentiostat, the scaffold was polarized at -0.6 $V_{Ag/AgCl}$ and MRSA biofilm was exposed to it for 6, 12, and 24 hr. As controls, non-polarized e-scaffolds were used. After treatment, the biofilm was scraped from the well bottom surface as well as from e-scaffold surface and these suspensions were vortexed and centrifuged (4000 rpm; 10 minutes). The cell pellet was resuspended in 1 ml of PBS and

serial dilutions were prepared. 100 μl of each dilution was spread-plated onto blood agar plates. Plates were then incubated at 37°C for 48 hr and colony forming units (CFU) were counted, with data reported as $\log_{10}$ CFU/cm^2.

Live/Dead Staining Assay: Freshly grown MRSA USA100 biofilms in 6-well glass-bottom plates were exposed to e-scaffold treatment for 6, 12, and 24 hr. As controls, non-polorized e-scaffolds were used. After exposure, live/dead staining assay was performed to assess cell membrane integrity of bacterial cells, using the BacLight Bacterial Viability Kit (L7007, Molecular Probes, Invitrogen, OR). After e-scaffold treatment, the biofilm was resuspended in 500 μl of 1X PBS. The biofilm samples were then stained with mixture of SYTO 9 and propidium iodide dyes (mixed according to te manufacturer's protocol) and incubated at room temperature for 15 min. The biofilm was viewed under confocal laser scanning microscope (Ziess LSM 780) at 400X magnification with the following filters: STYO 9: excitation/emission, 485/510 nm; propidium iodide: excitation/emission, 485/630 nm. For measuring biofilm depth, z-stack images were acquired at 1 μm intervals. At least 5 regions were scanned for each sample. Preliminary image analysis was performed using Zen 2 (V 2.0, Carl Zeiss Microscopy GmbH) software and final fluorescent biofilm z-stack images were then merged in ImageJ software (NIH).

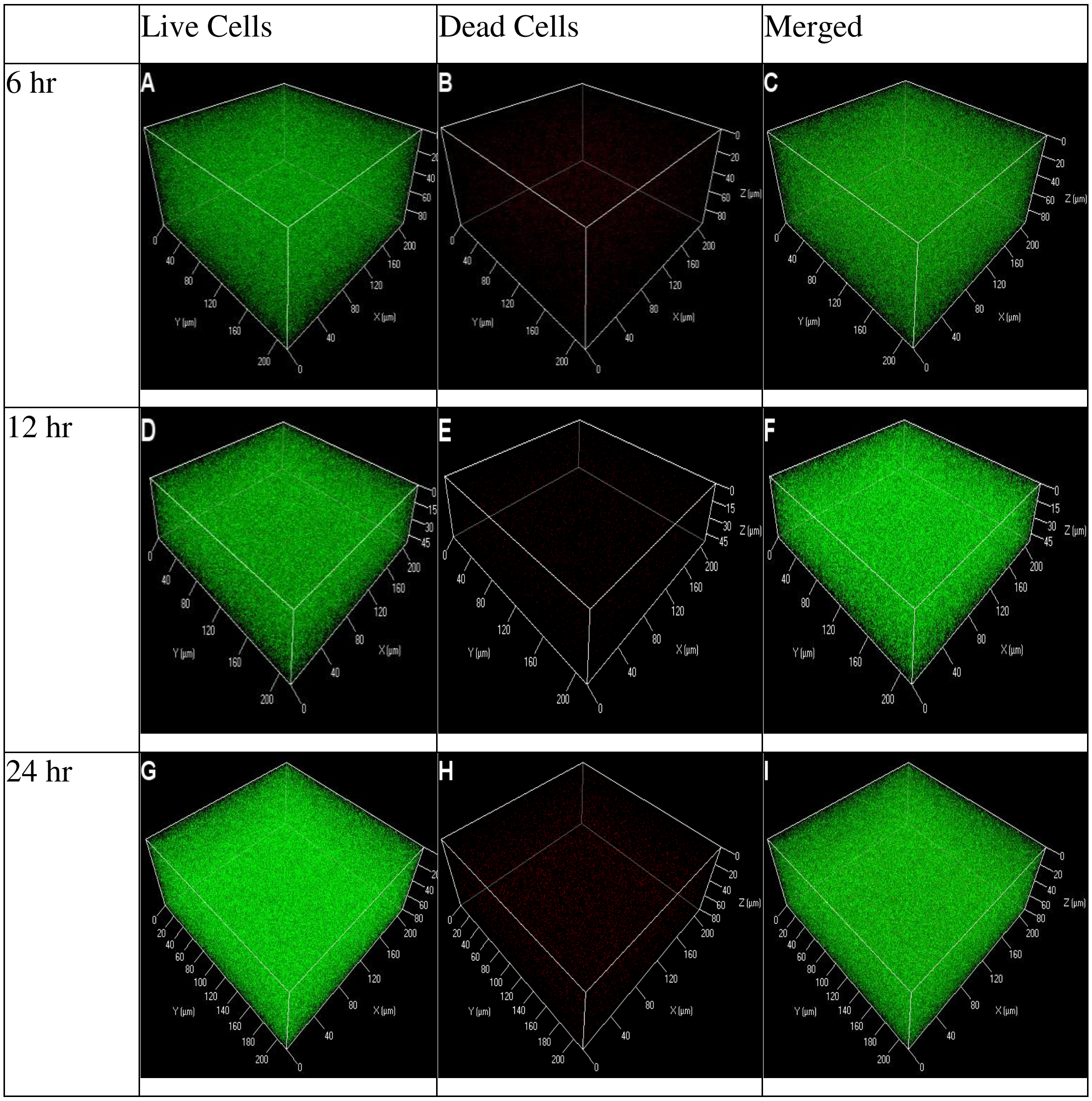

Figure S1. Live/Dead staining assay. MRSA USA100 biofilms were stained with a mixture of SYTO 9 and propidium iodide dyes. MRSA USA100 control biofilms (no exposure to e-scaffold treatment) at 6, 12, and 24 hours (hr). Live cells in biofilms appear green and dead cells appear red. Magnification: 400X

Intracellular ATP Assay: Freshly grown MRSA USA100 biofilms in 6-well glass-bottom plates were exposed to e-scaffold treatment for 6 hr, 12 hr, and 24 hr. As controls, non-polarized e-scaffolds were used. After exposure, the biofilm was scraped from the

glass-well surface as well as from e-scaffold surface and these suspensions were vortexed and centrifuged (4000 rpm; 10 minutes). The cell pellet was resuspended in 500 µl of 1X PBS. Finally, the intracellular ATP assay was performed utilizing the BacTiter-Glo kit (Promega, Madison, WI) per the manufacturer's protocol. The plate was read in a luminometer micro-plate reader (Synergy Hybrid H1, Biotek), with results obtained expressed in relative luminescent units.

Statistical Analysis: Statistical analysis was performed using Graphpad prism (V 7.0, CA, USA). Experiments were conducted in triplicate and data expressed as mean±SD. ANOVA was performed to determine significant statistical differences between groups and post hoc group comparisons were calculated through Tukey's post-tests. p-values <0.05 were considered statistically significant.
